# Skeletal Muscle MicroRNAs: Their Diagnostic and Therapeutic Potential in Human Muscle Diseases

**DOI:** 10.3233/JND-140058

**Published:** 2015

**Authors:** Matthew S. Alexander, Louis M. Kunkel

**Affiliations:** a Division of Genetics and Genomics at Boston Children's Hospital, Boston, MA, USA; b Department of Pediatrics and Genetics at Harvard Medical School, Boston, MA, USA; c The Stem Cell Program at Boston Children's Hospital, Boston, MA, USA; d The Manton Center for Orphan Disease Research at Boston Children's Hospital, Boston, MA, USA; e Harvard Stem Cell Institute, Cambridge, MA, USA

**Keywords:** MicroRNA, skeletal muscle, muscle disease, dystrophy, biomarker, therapy

## Abstract

MicroRNAs (miRNAs) are small 21–24 nucleotide RNAs that are capable of regulating multiple signaling pathways across multiple tissues. MicroRNAs are dynamically regulated and change in expression levels during periods of early development, tissue regeneration, cancer, and various other disease states. Recently, microRNAs have been isolated from whole serum and muscle biopsies to identify unique diagnostic signatures for specific neuromuscular disease states. Functional studies of microRNAs in cell lines and animal models of neuromuscular diseases have elucidated their importance in contributing to neuromuscular disease progression and pathologies. The ability of microRNAs to alter the expression of an entire signaling pathway opens up their unique ability to be used as potential therapeutic entry points for the treatment of disease. Here, we will review the recent findings of key microRNAs and their dysregulation in various neuromuscular diseases. Additionally, we will highlight the current strategies being used to regulate the expression of key microRNAs as they have become important players in the clinical treatment of some of the neuromuscular diseases.

## Introduction

MicroRNAs were first identified as anti-sense RNAs in *C. elegans* that were capable of regulating the expression levels of proteins by directly binding to the 3'UTR (untranslated region) of their complementary mRNA target [1]. Mammalian microRNAs were soon quickly identified and classified based on their sequence homology with known *C. elegans* microRNAs [2–4]. Improvements in whole-genome, next-generation sequencing technologies, and large scale transcriptome profiling have led to a subsequent identification and classification of mammalian microRNAs at a rapid rate starting in the mid to late-2000 s [5, 6]. Parallel computational methods and software algorithms have been developed which allow for the prediction of microRNA target genes in mammals [7–9].

Early gain and loss-of-function microRNA experiments demonstrated that microRNAs have significant roles in the regulation of protein function in many mammalian processes such as cancer, immune regulation, and cardiac function [10–13]. Interestingly, several of the early microRNAs identified as having significant roles in cardiac development and function, also had significant enrichment in expression in skeletal muscles [14, 15]. Large-scale microRNA microarray platforms identified microRNAs that were uniquely expressed and dysregulated in a variety of different human neuromuscular diseases [16]. Serum microRNA profiling of various neuromuscular diseases also revealed a similar dysregulation of particular microRNAs, and a unique diagnostic signature dependent on the mutation of the specific gene such as dystrophin [17, 18].

### MicroRNAs as biomarkers and therapeutic entry points for treatment of muscle disease

**Fig. 1:**
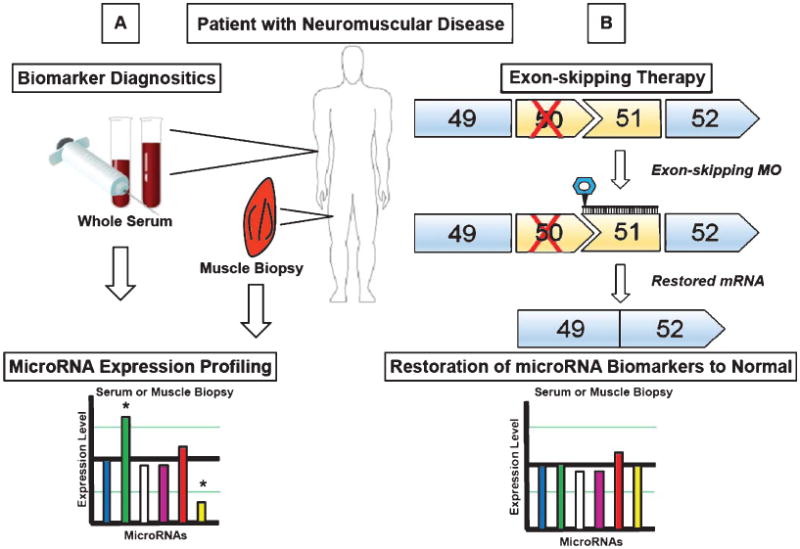
MicroRNAs can be isolated from serum and diseased muscles as diagnostic and quantitative biomarkers. Schematic showing the potential for the use of microRNAs both as diagnostic biomarkers (A) and quantitative biomarkers (B) in neuromuscular disease. A. Serum or a muscle biopsy can be taken from a patient with a known or undiagnosed neuromuscular disease. The sample is then analyzed for expression level and compared with known expression levels of specific microRNAs known to be dysregulated in particular neuromuscular diseases. B. An exon-skipping morpholino is used as a therapeutic intervention strategy to bypass the DNA deletion of exon 50 (red letter X) of protein-encoding gene (e.g. dystrophin). The exon-skipping morpholino skips over exon 51 to restore the correct reading frame of the mRNA transcript. The resulting mRNA transcript is spliced together to restore function and/or reading frame of the mature mRNA when it will be translated into a mature protein by the ribosomal machinery. The microRNA levels are used as a non-evasive biomarker, and the microRNA biosignature is monitored for restoration to that of normal healthy muscle control expression levels.

An area of microRNA biology that has gained a lot of recent attention is the ability of microRNAs to circulate in the bloodstream via exosomes. MicroRNAs can be secreted from one tissue type and be transported to more distal tissues after being incorporated in multi-vesicular bodies derived from the plasma membrane of cells. It remains unclear whether or not the distal (or target) tissues that incorporate these microRNA-containing exosomes function to inhibit target mRNAs, but this remains an active area of research. Nevertheless, the isolation and identification of specific miRNAs from these exosomes can reveal a significant shift towards a diseased state (Fig. 1A).

Serum profiling of microRNAs from neuromuscular diseases has revealed that specific microRNAs are indeed dysregulated in expression levels in a disease-dependent fashion (Table 1). MicroRNAs isolated from the serum of patients with Duchenne muscular dystrophy revealed that specific muscle-enriched microRNAs were significantly altered in expression dependent on the progression of the dystrophic disease pathology [18]. Similar results were observed in dystrophic *mdx* mouse muscles, which revealed that one particular muscle-enriched microRNA, miR-206, was significantly increased in expression levels when compared with normal mouse muscles [19].

MicroRNAs represent a unique therapeutic entry point for disease as a single microRNA can regulate multiple signaling pathways rather than the classical one gene, one target approach. Thus, several pharmaceutical and biotech companies have begun to develop microRNA-based therapies for the treatment of disease in addition to using them as clinical biomarkers of disease states [20, 21]. Recently, a miR-122 locked nucleic acid (LNA)-inhibitor, Miravirsen (SPC3649), has shown therapeutic efficacy and benefit in blocking hepatitis C (HCV) viral replication in phase 2a clinical trials [22]. Additionally, it has been demonstrated that manipulation of microRNAs using mouse models of neuromuscular diseases can ameliorate some of the disease pathologies associated with changes in the expression levels of specific microRNAs [23–26]. Given the broad reaching applications of microRNAs as both molecular biomarkers and therapeutic entry points, it is likely that microRNAs will become key mediators in the identification and treatment of patients with neuromuscular diseases. For example, a patient with an identified neuromuscular pathology and showing symptoms of a particular type of neuromuscular disease, but no mutation in the causative gene for that particular disease may have their blood drawn and microRNA profiled for diagnostic purposes to identify or exclude a particular disease with a defined microRNA signature (Fig. 1A). One could also apply an appropriate therapeutic course of intervention to a particular group of patients and use the microRNA signature as a biomarker to see if the dysregulated microRNAs return to more normal levels (Fig. 1B).

### MicroRNAs are dynamically regulated in many muscle diseases

#### Duchenne muscular dystrophy (DMD)

One of the most studied neuromuscular diseases with significant microRNA dysregulation is Duchenne muscular dystrophy (DMD) [27]. Patients with mutations in the *DMD* gene that result in the loss of the large dystrophin protein isoform (Dp427) show loss of ambulation, severe muscle degeneration, and heart disease [28]. MicroRNA microarray profiling of dystrophin-deficient muscles from human patient biopsies revealed a unique signature of dysregulated muscle-enriched microRNAs when compared to other muscular dystrophies [16]. Follow-up studies in muscles of dystrophin-deficient mdx mice demonstrated that many microRNAs that regulate nNOS signaling, with a particular dysregulation of miR-1, miR-133a/b, and miR-206 (also referred to as “myomiRs”), were significantly altered by the loss of a functional dystrophin protein [29, 30]. MyomiRs (a term coined by combining myo/muscle and miR/microRNA) was used to originally describe three microRNAs (miR-1, miR-133a/b, and miR-206) that showed enriched expression in heart and skeletal muscles; but has since expanded from its original definition to include several additional microRNAs that are strongly expressed in muscle lineages [31, 32]. Profiling of human DMD patient myoblasts confirmed the dysregulation of miR-1, but also found a significant dysregulation in the expression of miR-29a both of which regulate a Dystrophin-nNOS-Hdac2 pathway [33]. Serum profiling of human patients revealed that the three muscle-enriched myomiR microRNAs were also dysregulated in both human patients and mdx mice [17, 18, 34, 35]. MicroRNA expression profiling of the serum from the dystrophic CXMDJ canine dystrophin-deficient model also showed a dysregulation of miR-1, miR-133a, and miR-206 [36]. Another recent study of serum obtained from DMD boys demonstrated that in addition to the three myomiRs (miR-1, miR-133a/b, and miR-206) being increased in expression, two other muscle-enriched microRNAs, miR-208b and miR-499 were also increased in expression [37] (Table 1). Another microRNA, miR-31, was shown to be significantly increased in DMD muscle, and might play a role in normal muscles in the regulation of the dystrophin protein levels via binding to its 3'UTR [38]. (Table 1). A more broadly expressed microRNA, miR-199a, was also shown to be induced in DMD muscle biopsies due to a transcriptional activation of its promoter via the myogenic factor serum response factor (SRF) [39]. It has also been demonstrated that the muscle-enriched microRNA, miR-486, is significantly decreased in expression in DMD patient muscle biopsies and myoblast cell lines, but not in the milder Becker muscular dystrophy (BMD) in which a partially functional dystrophin protein is produced [40]. Thus, it can be concluded that many microRNAs that are enriched in expression in skeletal muscle appear to be strongly dysregulated in Duchenne muscular dystrophy.

The functional roles of these muscle-enriched dysregulated DMD microRNAs (or “dystromiRs” as they are sometimes referred to as), lead to functional studies in mouse and muscle cell culture models [18]. These microRNAs (miR-1, miR-133a/b, and miR-206) were first given the classification as “dystromiRs” as potential diagnostic markers due to their dysregulation in dystrophin-deficient mdx mouse and human DMD patient skeletal muscles [17]. Global loss of both copies of miR-1 (miR-1-1 and miR-1-2) in mice revealed an essential function for miR-1 in postnatal cardiac conduction function, sarcomere formation, and activation of smooth muscle gene expression [41, 42]. Similar global deletion of both copies of miR-133a (miR-133a-1 and miR-133a-2) revealed an essential role for miR-133a in postnatal cardiac function, normal cardiomyocyte proliferation, and activation of SRF-dependent smooth muscle gene transcription [43]. Compound deletions of miR-1-1/miR-133a-2 and miR-1-2/miR-133a-1, which in mammals are clustered and transcribed at the same genomic locus, revealed a role for these microRNAs as a regulator of smooth muscle gene transcription via suppression of the SRF cofactor myocardin [44]. The muscle enriched microRNA, miR-206, has been shown to be overexpressed in dystrophic and regenerating skeletal muscle samples along with serum from dystrophic patients and animals [16, 18, 45, 46]. Surprisingly, mice lacking miR-206 showed no overt skeletal muscle or cardiac phenotypes, which has led to the speculation that another microRNA may be playing a compensatory role in its absence [47].

A significant number of muscle diseases including DMD have elevated levels of microRNAs associated with fibrosis. One induced microRNA greatly associated with the fibrotic response in skeletal muscle disease is miR-21. MicroRNA-21 activation is strongly correlated with proliferation of fibroblasts and activation of TGFβ signaling in several models of fibrotic-associated diseases such as idiopathic pulmonary fibrosis (IPF) [48]. MicroRNA-21 is strongly induced in expression in DMD biopsies and is thought to be regulated by plasminogen activator inhibitor-1 (PAI-1) [16, 26]. PAI-1 is a key regulator of the extracellular matrix (ECM) and fibrotic response in *mdx* mouse muscles [26, 49]. Interestingly, miR-21 global knockout mice are viable and have reduced tumorigenic capacity in several non-muscle cell cancers due to an induction of the inhibitors of the Ras/MEK/ERK signaling pathways that normally repressed by miR-21 function [50, 51]. MicroRNA-21 was also shown to be significantly increased in expression in rodent models of myocardial disease, and activate ERK/MAPK signaling pathways in cardiac fibroblasts [52]. However, it is important to note that miR-21 global knockout mice showed the same stress-dependent cardiac remodeling that occurred in wild type control mice, suggesting that miR-21 inhibitor molecules might not have significant beneficial effects on myocardial disease pathologies [53]. Additionally, endothelial cell-specific miR-21 knockout mice showed significant vasculature remodeling concomitant with a reduction of collagen and other ECM proteins [54]. Conversely, overexpression of miR-21 in gain-of-function mouse models results in a tissue-specific promotion tumorigenesis and cell proliferation [55]. Administration of TGFβ inhibitors in *mdx* mice subsequently blocks fibrosis and decreases miR-21 expression levels, thus making miR-21 both an important regulator of the dystrophic disease pathology and a useful biomarker for fibrotic response in muscle disease [56].

#### Facioscapulohumeral muscular dystrophy (FSHD)

Facioscapulohumeral muscular dystrophy (FSHD) is generally considered to be caused by the contraction of D4Z4 repeats subsequently leading to the activation of a transcription factor DUX4 in skeletal muscle [57, 58]. More recently mutations in the chromatin remodeling factor SMCHD1 have been shown to affect DUX4 expression and distinguish FSHD Type 1 from FSHD Type 2[59]. The dynamic regulation of the expression of DUX4 and its transcriptional regulation have highlighted significant dysregulation of microRNAs during FSHD disease progression [60, 61]. The overexpression of microRNA-411 in FSHD myoblasts has been implicated as a potential mechanism for the blocking of myogenic differentiation via directly suppressing YAF2 and YY1 transcriptional function [62]. Full transcriptome analysis of microRNAs dysregulated in FSHD myoblasts and serum from FSHD patients revealed a significant increase in expression of the muscle myomiRs (miR-1, miR-133a/b, miR-206) along with significant dysregulation of several other microRNAs [63, 64] (Table 1). Next-generation sequencing of FSHD myoblasts reported several additionally dysregulated microRNAs when compared with unaffected patient myoblasts [65] (Table 1).Additionally, a long-noncoding RNA (lncRNA)*DBE-T* has been implicated in the transcriptional activation of DUX4, and may also play a significant role in the promotion of the FSHD disease pathology [66]. Given the complexity of FSHD disease progression, epigenetic regulation by non-coding RNAs in FSHD muscles might offer an explanation for the unique dysregulation of myogenic and non-myogenic signaling pathways that occur in the FSHD disease state [67–69].

#### Limb girdle muscular dystrophies (LGMDs) and other neuromuscular diseases

Several other human neuromuscular diseases have strong etiopathologies associated with a dysregulation of microRNAs. The mouse model of laminin α2 chain (MDC1A) congenital muscular dystrophy showed significant alterations in expression levels of both the myomiRs and fibrosis-associated microRNAs [70]. LGMD2A is caused by a deficiency of the protease calpain-3 that results in muscle satellite cell defects, and a subsequent reduction in expression of the muscle-enriched myomiRs [71, 72]. Additionally, mouse models of LGMD2C, LGMD2D, and Emery-Dreifuss muscular dystrophy (EDMD) have revealed novel insights into the disease progression by identification of a unique microRNA diagnostic signatures throughout each diseases chronological progression [73]. Myotonic dystrophy is a multi-system disorder affecting skeletal muscle, brain, heart, and other organs commonly found in adults and is caused by either CTG (DM1; Myotonic dystrophy type 1) or CCTG (DM2; Myotonic dystrophy type 2) pathogenic repeat expansions of either DMPK (DM1) or ZNF9 (DM2) RNA transcripts [74, 75]. MicroRNA profiling of primary skeletal muscle myoblasts derived from DM1 and DM2 patients revealed unique diagnostic signatures of dysregulated microRNAs dependent on the type of myotonic dystrophy [76, 77] (Table 1). Several of these microRNA studies demonstrate the dynamic dysregulation of expression that can occur with various microRNAs that have known or unknown functions in skeletal muscle. One study of microRNAs dysregulated in DM2 patient myoblasts demonstrated that miR-221, a microRNA shown to decrease in expression in the differentiation of quail myoblasts was significantly increased in total expression level [77, 78] (Table 1). Conversely, microRNA-378a, a microRNA shown to be a regulator of metabolism and obesity, was significantly deceased in DM2 patient myoblasts [77, 79] (Table 1). Interestingly, in the hearts of DM1 and DM2 patients the pri-miR-1 stem loop expression level increases in overall expression; however, the mature miR-1 sequence is overall reduced in comparison with unaffected patient hearts [80]. Conversely, mature miR-1 is significantly increased in expression levels in DM1 patient primary myoblast cell lines when compared to unaffected control patient myoblast [76] (Table 1). The authors of the study showing decreased levels of miR-1 in DM1 and DM2 patient hearts (despite increased levels of pri-miR-1) demonstrate that miR-1 biogenesis is significantly altered due to the muscleblind protein (MBNL1) sequestration in the nucleus and dysregulation of microRNA interacting RNA-binding factors [80]. Thus, it is possible that there are different tissue-specific post-transcriptional regulators of the microRNA processing machinery might be the causative mechanism for such disparate results of microRNA dysregulation in separate muscle tissues. These studies highlight the principle that the dysregulation of microRNAs in various muscle diseases can yield unique diagnostic signatures that are highly dependent on the causative disease mutation and potentially have tissue-specific effects.

### Therapeutic inhibition of microRNAs in muscle diseases

**Fig. 2:**
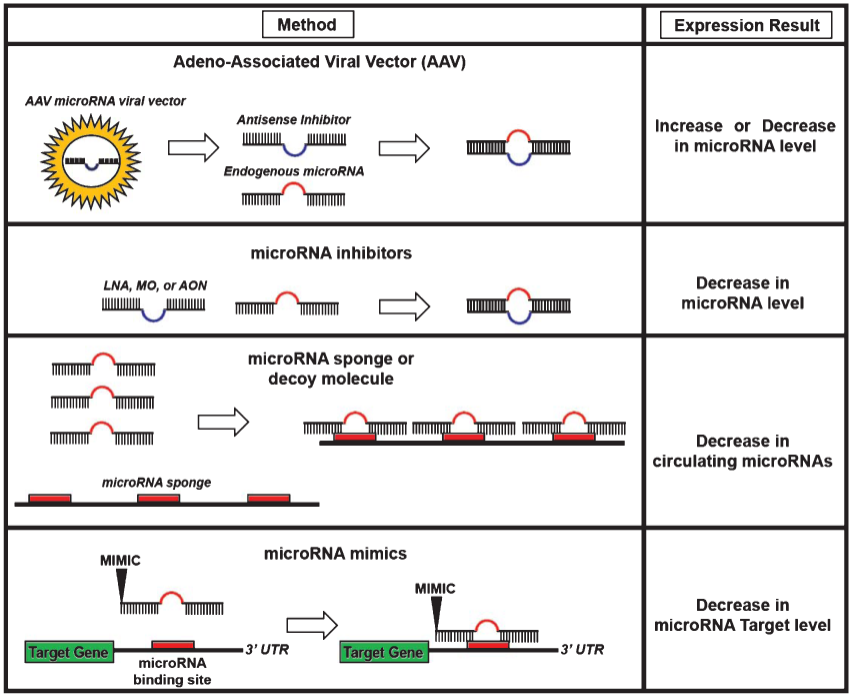
Strategies to manipulate expression levels of microRNAs for the treatment of neuromuscular diseases. Therapies using Adeno-Associated Viral vectors (AAV) delivery to increase or decrease the expression levels of a specific microRNA that could be used to treat either primary or secondary consequences of the neuromuscular disease mutations and/or its disease-associated pathological symptoms. Synthetic approaches involving microRNA inhibitors (LNAs, MOs, or other AON molecules; blue seed loop) or microRNA sponges (or other “decoy molecules”) contain microRNA binding sites (red rectangles) and might be used to inhibit microRNA function via direct antisense inhibition thereby reducing the levels of endogenous microRNAs (red seed loop) in the serum or tissue. MicroRNA sponges or decoy molecules can be used to remove the amount of circulating or (“unbound”) microRNAs in a given tissue or from serum. Other synthetic molecules, such as microRNA mimics (red seed loop; MIMIC), might be used to mimic the function of endogenous microRNAs thereby suppressing the microRNA's intended mRNA target gene.

Inhibition of microRNAs *in vivo* can be achieved via injection or oral delivery of anti-sense 2′-O-methyl oligonucleotide inhibitors, antagomiRs, or locked nucleic acids (LNAs; a RNA-based molecule whose ribose moiety is modified with an extra bridge connecting the 2′ oxygen and 4′ carbon) that target either the mature miRNA or the pri-miR precursor microRNA [81–85]. These antisense approaches work by blocking the target microRNA via direct binding, thereby inhibiting the microRNA from binding to the 3'UTR of target mRNAs (Fig. 2). Additional strategies include the use of multimerized microRNA complementary DNA sequences that act as “sponges” to block microRNA function by increasing the amount of bound microRNA to its complementary sequence [86]. It has already been shown that microRNA sponges can block cancer microRNAs (or “oncomiRs) from regulating their mRNA targets, thus having profound effects on tumor propagation and metastasis [87, 88]. Several groups have multimerized microRNA target sequences as another means of reducing the levels of unbound or circulating microRNAs. One study generated an AAV overexpressing miR-206 sponge consisting of multimerized miR-206 binding sites, and demonstrated successful inhibition of miR-206 levels following injection into mice [89]. Furthermore, another group identified a small molecule inhibitor of the three myomiR function *in vitro* [90]. Inhibition of specific microRNAs has already shown therapeutic benefits in mouse models of cardiac hypertrophy [91, 92]. Thus, novel inhibitors (antisense or other methods) of microRNAs that are induced in specific neuromuscular diseases might hold promise in ameliorating specific aspects of the disease progression.

### Strategies to overexpress microRNAs in muscle

Stable, long-term overexpression of specific-microRNAs in mammals has been demonstrated using Adeno-Associated Viral Vectors (AAVs) for several different diseases [93–95]. However, high-doses of microRNA-overexpressing AAV viral particles had been previously shown to induce liver failure due to a saturation of the microRNA/shRNA processing machinery, thus making the virus delivery strategy, dosage, and serotype important in reducing any liver toxicity [96]. AAV serotypes AAV-6, -8, and -9 have been shown to be effective in the delivery of micro-dystrophin and/or other constructs to dystrophin-deficient skeletal and heart muscles [97–100]. Femoral AAV delivery of miR-196a into a mouse model of spinal and bulbar muscular atrophy (SBMA) was effective and therapeutically efficacious in targeting CELF2, a CUG-repeat binding protein that causes RNA toxicity by trapping it in skeletal muscles [24]. Other approaches such as the AAV over-expression of miR-669a in β-sarcoglycan (Sgcb)-null hearts showed long-term, and potent affects in restoring sarcomere organization and cardiac function [95]. More recently, efforts have been made to generate synthetic microRNA mimics with enhanced stability and reduced toxicity for *in vivo* animal use [101]. However, there is little known about the long-term effects and potency of these synthetic microRNA mimics, and more work is required to optimize their delivery in to muscle tissues.

## Conclusions

There have been several recent studies that have attempted to manipulate the expression levels of microRNAs and more-importantly their mRNA targets in order to ameliorate neuromuscular disease pathologies. To suppress expression of the toxic DUX4 protein in FSHD, AAV vectors carrying artificial microRNA-based DUX4 open reading frame (ORF) inhibitors showed efficacy in a DUX4 overexpressing mouse model [102]. Manipulation of expression of the muscle-enriched microRNAs miR-206 and miR-486 in mouse models of DMD showed benefits in reducing fibrosis, promoting muscle regeneration, and improving overall muscle physiological strength [23, 25]. It is unclear whether or not these and other muscle-enriched microRNAs would have similar benefits in other mouse models of muscular dystrophy. As of to date, no specific human mutation in a microRNA sequence has been directly linked to a neuromuscular disease; however, there are some examples of microRNAs that when genetically manipulated result in muscle phenotypes similar to those found in human patients with neuromuscular diseases. MicroRNA-133a mutant mice develop centronuclear myopathy (CNM)-like symptoms due to miR-133a's direct regulation of the dynamin2 (DNM2) transcript [103]. Additionally, a naturally-occurring SNP mutation in the 3'UTR of the Myostatin (GDF8) gene generated a novel miR-1/206 binding site resulting in both a dramatic decrease in myostatin protein and a consequential increase in muscle size [104]. It is likely that with advances in next-generation sequencing technologies additional patient mutations in UTR's and perhaps even microRNA genomic sequences might directly be causative for neuromuscular diseases.

MicroRNAs may likely be used as biomarkers for testing the efficacy of a treatment for neuromuscular disease. For example, phosphorodiamidate morpholino oligonucleotide (PMO)-mediated dystrophin restoration therapy in *mdx* mice was able to correct the dysregulation of the myomiRs (miR-1, -133a/b, -206) to normal wild type levels in mouse serum indicating the dynamic nature of microRNA expression in neuromuscular disease [34]. Indeed, measurement of the disease progression severities such as the trait of loss of ambulation amongst DMD patients, may be quantitatively measured using serum microRNA biosignatures as useful predictor of drug benefits in DMD treatment patient cohorts [46]. With a renewed emphasis towards non-invasive clinical biomarkers of neuromuscular disease therapies, microRNAs are an ideal biomarker to quantitatively measure the effectiveness of novel drug therapies (Fig. 1B). In conclusion, microRNAs are key players in the neuromuscular diseases, and can be exploited as therapeutic entry points for the treatment of disease due to their dynamic regulation of many cellular functions.

## Figures and Tables

**Table 1:** Several microRNAs known and validated as dysregulated in expression levels in different common neuromuscular disease. MicroRNA name, neuromuscular disease, sample tested, expression change (compared to unaffected controls), and reference are listed. For the myomiRs (miR-1, -133a/b, and 206) dysregulation was shown using serum from patients, except for DM1 (patient skeletal muscle biopsies). Note, only those publications using quantitative measurements (i.e. not only microRNA microarray fold changes such as real time quantitative PCR) are represented in the table

microRNA	Neuromuscular disease	Sample	Expression change	Reference(s)
miR-1	DMD, BMD, FSHD, DM1	serum, skeletal muscle biopsies (DM1)	increased	18, 37, 46, 64, 73, 76
miR-21	MM, IBM PM	skeletal muscle biopsies	increased	16
miR-29b/c	DM1	skeletal muscle biopsies	decreased	76
miR-31	DMD	skeletal muscle biopsies	increased	38
miR-33	DM1	skeletal muscle biopsies	decreased	76
miR-34a/b/c	DM2	skeletal muscle biopsies	increased	77
miR-125b	DM2	skeletal muscle biopsies	decreased	77
miR-133a/b	DMD, BMD	serum, skeletal muscle biopsies (DM1)	increased	18, 37, 46, 64
miR-146b	DM2	skeletal muscle biopsies	increased	77
miR-193a/b	DM2	skeletal muscle biopsies	decreased	77
miR-199a	DMD	skeletal muscle biopsies	increased	39
miR-206	DMD, BMD, DM1	serum, skeletal muscle biopsies (DM1)	increased	18, 37, 46, 64, 73
miR-208a/b	DMD; DM2	serum, skeletal muscle biopsies (DM2)	increased	37, 77
miR-221	DM2	skeletal muscle biopsies	increased	77
miR-335	DM1	skeletal muscle biopsies	increased	76
miR-378a	DMD; DM2	serum; skeletal muscle biopsies	increased (DMD); decreased (DM2)	73, 77
miR-381	DM2	skeletal muscle biopsies	increased	77
miR-411	FSHD	skeletal muscle myoblasts	increased	62
miR-486	DMD	skeletal muscle biopsies	decreased	40
miR-499	DMD	serum	increased	37

## Abbreviations

AAV: Adeno-associated viral vector
AON: Antisense oligonucleotide
BMD: Becker muscular dystrophy
CMD: Congenital muscular dystrophy
CXMDJ: Canine X-linked muscular dystrophy in Japan
DM1: Myotonic dystrophy type 1
DM2: Myotonic dystrophy type 2
DMD: Duchenne muscular dystrophy
EDMD: Emery-Dreifuss muscular dystrophy
FSHD: Facioscapulohumeral muscular dystrophy
IBM: Inclusion body myositis
LNA: Locked nucleic acid
lncRNA: Long, non-coding RNA
miR: MicroRNA
MM: Miyoshi myopathy
MO: Morpholino
NM: Nemaline myopathy
ORF: Open reading frame
PM: Polymyositis
PMO: Phosphorodiamidate morpholino oligonucleotide
shRNA: Short hairpin RNA
SRF: Serum response factor
UTR: Untranslated region

## References

[1] Lee RC, Feinbaum RL, Ambros V (1993). The C. elegans heterochronic gene lin-4 encodes small RNAs with antisense complementarity to lin-14. Cell.

[2] Lagos-Quintana M (2002). Identification of Tissue-Specific MicroRNAs from Mouse. Current Biology.

[3] Lagos-Quintana M (2001). Identification of Novel Genes Coding for Small Expressed RNAs. Science.

[4] Lagos-Quintana M (2003). New microRNAs from mouse and human. RNA.

[5] Bartel DP (2004). MicroRNAs: Genomics, Biogenesis, Mechanism, and Function. Cell.

[6] Li L (2010). Computational approaches for microRNA studies: a review. Mammalian Genome.

[7] Rajewsky N, Socci ND (2004). Computational identification of microRNA targets. Developmental Biology.

[8] Kiriakidou M (2004). A combined computational-experimental approach predicts human microRNA targets. Genes & Development.

[9] Bartel DP (2009). MicroRNAs: Target Recognition and Regulatory Functions. Cell.

[10] Lim LP (2005). Microarray analysis shows that some microRNAs downregulate large numbers of target mRNAs. Nature.

[11] Thai TH (2007). Regulation of the Germinal Center Response by MicroRNA-155. Science.

[12] Ventura A (2008). Targeted Deletion Reveals Essential and Overlapping Functions of the miR-17∼92 Family of miRNA Clusters. Cell.

[13] van Rooij E (2007). Control of Stress-Dependent Cardiac Growth and Gene Expression by a MicroRNA. Science.

[14] Zhao Y, Samal E, Srivastava D (2005). Serum response factor regulates a muscle-specific microRNA that targets Hand2 during cardiogenesis. Nature.

[15] Chen JF (2006). The role of microRNA-1 and microRNA-133 in skeletal muscle proliferation and differentiation. Nat Genet.

[16] Eisenberg I (2007). Distinctive patterns of microRNA expression in primary muscular disorders. Proceedings of the National Academy of Sciences.

[17] Cacchiarelli D (2011). miRNAs as serum biomarkers for Duchenne muscular dystrophy. EMBO Molecular Medicine.

[18] Zaharieva IT (2013). Dystromirs as Serum Biomarkers for Monitoring the Disease Severity in Duchenne Muscular Dystrophy. PLoS ONE.

[19] Roberts TC (2013). Extracellular microRNAs are dynamic non-vesicular biomarkers of muscle turnover. Nucleic Acids Research.

[20] Calway T, Kim GH (2014). Harnessing the Therapeutic Potential of MicroRNAs for Cardiovascular Disease. Journal of Cardiovascular Pharmacology and Therapeutics.

[21] Monroig P d C Small molecule compounds targeting miRNAs for cancer therapy. Advanced Drug Delivery Reviews.

[22] Janssen HLA (2013). Treatment of HCV Infection by Targeting MicroRNA. New England Journal of Medicine.

[23] Liu N (2012). microRNA-206 promotes skeletal muscle regeneration and delays progression of Duchenne muscular dystrophy in mice. The Journal of Clinical Investigation.

[24] Miyazaki Y (2012). Viral delivery of miR-196a ameliorates the SBMA phenotype via the silencing of CELF2. Nat Med.

[25] Alexander MS (2014). MicroRNA-486–dependent modulation of DOCK3/PTEN/AKT signaling pathways improves muscular dystrophy–associated symptoms. The Journal of Clinical Investigation.

[26] Ardite E (2012). PAI-1-regulated miR-21 defines a novel age-associated fibrogenic pathway in muscular dystrophy. The Journal of Cell Biology.

[27] Sharma M (2014). Mega roles of microRNAs in regulation of skeletal muscle health and disease. Frontiers in Physiology.

[28] Hoffman EP, Brown RH, Kunkel LM (1987). Dystrophin: the protein product of the Duchenne muscular dystrophy locus. Cell.

[29] Cacchiarelli D (2010). MicroRNAs Involved in Molecular Circuitries Relevant for the Duchenne Muscular Dystrophy Pathogenesis Are Controlled by the Dystrophin/nNOS Pathway. Cell metabolism.

[30] Townley-Tilson WHD, Callis TE, Wang D (2010). MicroRNAs 1. 133, and 206: Critical factors of skeletal and cardiac muscle development, function, and disease. The International Journal of Biochemistry & Cell Biology.

[31] Sempere L (2004). Expression profiling of mammalian microRNAs uncovers a subset of brain-expressed microR-NAs with possible roles in murine and human neuronal differentiation. Genome Biology.

[32] McCarthy JJ (2008). MicroRNA-206: The skeletal muscle-specific myomiR. Biochimica et Biophysica Acta (BBA) – Gene Regulatory Mechanisms.

[33] Cazzella V (2012). Exon 45 Skipping Through U1-snRNA Antisense Molecules Recovers the Dys-nNOS Pathway and Muscle Differentiation in Human DMD Myoblasts. Mol Ther.

[34] Roberts TC (2012). Expression Analysis in Multiple Muscle Groups and Serum Reveals Complexity in the MicroRNA Transcriptome of the mdx Mouse with Implications for Therapy. Mol Ther Nucleic Acids.

[35] Greco S (2009). Common micro-RNA signature in skeletal muscle damage and regeneration induced by Duchenne muscular dystrophy and acute ischemia. The FASEB Journal.

[36] Mizuno H (2011). Identification of Muscle-Specific MicroRNAs in Serum of Muscular Dystrophy Animal Models: Promising Novel Blood-Based Markers for Muscular Dystrophy. PLoS ONE.

[37] Li X (2014). Circulating Muscle-specific miRNAs in Duchenne Muscular Dystrophy Patients. Mol Ther Nucleic Acids.

[38] Cacchiarelli D (2011). miR-31 modulates dystrophin expression: new implications for Duchenne muscular dystrophy therapy. EMBO Rep.

[39] Alexander MS (2013). MicroRNA-199a is induced in dystrophic muscle and affects WNT signaling, cell proliferation, and myogenic differentiation. Cell Death Differ.

[40] Alexander M (2011). Regulation of DMD pathology by an ankyrin-encoded miRNA. Skeletal Muscle.

[41] Wei Y (2014). Multifaceted roles of miR-1s in repressing the fetal gene program in the heart. Cell Res.

[42] Heidersbach A (2013). microRNA-1 regulates sarcomere formation and suppresses smooth muscle gene expression in the mammalian heart. Elife.

[43] Liu N (2008). microRNA-133a regulates cardiomyocyte proliferation and suppresses smooth muscle gene expression in the heart. Genes & Development.

[44] Wystub K (2013). miR-1/133a Clusters Cooperatively Specify the Cardiomyogenic Lineage by Adjustment of Myocardin Levels during Embryonic Heart Development. PLoS Genet.

[45] McCarthy JJ, Esser KA, Andrade FH (2007). MicroRNA-206 is overexpressed in the diaphragm but not the hindlimb muscle of mdx mouse. Am J Physiol Cell Physiol.

[46] Hu J (2014). Serum miR-206 and other muscle-specific microRNAs as non-invasive biomarkers for Duchenne muscular dystrophy. Journal of Neurochemistry.

[47] Williams AH (2009). MicroRNA-206 Delays ALS Progression and Promotes Regeneration of Neuromuscular Synapses in Mice. Science.

[48] Liu G (2010). miR-21 mediates fibrogenic activation of pulmonary fibroblasts and lung fibrosis. The Journal of Experimental Medicine.

[49] Ghosh AK, Vaughan DE (2012). PAI-1 in tissue fibrosis. Journal of Cellular Physiology.

[50] Hatley ME (2010). Modulation of K-Ras-Dependent Lung Tumorigenesis by MicroRNA-21. Cancer Cell.

[51] Ma X (2011). Loss of the miR-21 allele elevates the expression of its target genes and reduces tumorigenesis. Proceedings of the National Academy of Sciences.

[52] Thum T (2008). MicroRNA-21 contributes to myocardial disease by stimulating MAP kinase signalling in fibroblasts. Nature.

[53] Patrick DM (2010). Stress-dependent cardiac remodeling occurs in the absence of microRNA-21 in mice. The Journal of Clinical Investigation.

[54] Zhang XY (2013). Induction of Thoracic Aortic Remodeling by Endothelial-Specific Deletion of MicroRNA-21 in Mice. PLoS ONE.

[55] Medina PP, Nolde M, Slack FJ (2010). OncomiR addiction in an in vivo model of microRNA-21-induced pre-B-cell lymphoma. Nature.

[56] Acuña MJ (2014). Restoration of muscle strength in dystrophic muscle by angiotensin-1-7 through inhibition of TGF-β signalling. Human Molecular Genetics.

[57] Deutekom JCTV (1993). FSHD associated DNA rearrangements are due to deletions of integral copies of a 3.2kb tandemly repeated unit. Human Molecular Genetics.

[58] Hewitt JE (1994). Analysis of the tandem repeat locus D4Z4 associated with facioscapulohumeral muscular dystropothhy. Human Molecular Genetics.

[59] Lemmers RJLF (2012). Digenic inheritance of an SMCHD1 mutation and an FSHD-permissive D4Z4 allele causes facioscapulohumeral muscular dystrophy type 2. Nat Genet.

[60] Cheli S (2011). Expression Profiling of FSHD-1 and FSHD-2 Cells during Myogenic Differentiation Evidences Common and Distinctive Gene Dysregulation Patterns. PLoS ONE.

[61] Winokur ST (2003). Expression profiling of FSHD muscle supports a defect in specific stages of myogenic differentiation. Human Molecular Genetics.

[62] Harafuji N (2013). miR-411 is up-regulated in FSHD myoblasts and suppresses myogenic factors. Orphanet Journal of Rare Diseases.

[63] Dmitriev P (2013). Defective Regulation of MicroRNA Target Genes in Myoblasts from Facioscapulohumeral Dystrophy Patients. Journal of Biological Chemistry.

[64] Matsuzaka Y (2014). Three novel serum biomarkers, miR-1, miR-133a, and miR-206 for Limb-girdle muscular dystrophy, Facioscapulohumeral muscular dystrophy, and Becker muscular dystrophy. Environmental Health and Preventive Medicine.

[65] Colangelo V (2014). Next-Generation Sequencing Analysis of MiRNA Expression in Control and FSHD Myogenesis. PLoS ONE.

[66] Cabianca Daphne S (2012). A Long ncRNA Links Copy Number Variation to a Polycomb/Trithorax Epigenetic Switch in FSHD Muscular Dystrophy. Cell.

[67] Tsumagari K (2011). Gene expression during normal and FSHD myogenesis. BMC Medical Genomics.

[68] Yao Z (2014). DUX4-induced gene expression is the major molecular signature in FSHD skeletal muscle. Human Molecular Genetics.

[69] Vanderplanck C (2011). The FSHD Atrophic Myotube Phenotype Is Caused by DUX4 Expression. PLoS ONE.

[70] Holmberg J (2014). Laminin α2 chain-deficiency is associated with microRNA deregulation in skeletal muscle and plasma. Frontiers in Aging Neuroscience.

[71] Rosales XQ (2013). Impaired regeneration in LGMD2A supported by increased PAX7-positive satellite cell content and muscle-specific microrna dysregulation. Muscle & Nerve.

[72] Richard I (1995). Mutations in the proteolytic enzyme calpain 3 cause limb-girdle muscular dystrophy type 2A. Cell.

[73] Vignier N (2013). Distinctive Serum miRNA Profile in Mouse Models of Striated Muscular Pathologies. PLoS ONE.

[74] Liquori CL (2001). Myotonic Dystrophy Type 2 Caused by a CCTG Expansion in Intron 1 of ZNF9. Science.

[75] Brook JD (1992). Molecular basis of myotonic dystrophy: Expansion of a trinucleotide (CTG) repeat at the 3′ end of a transcript encoding a protein kinase family member. Cell.

[76] Perbellini R (2011). Dysregulation and cellular mislocalization of specific miRNAs in myotonic dystrophy type 1. Neuromuscular Disorders.

[77] Greco S (2012). Deregulated MicroRNAs in Myotonic Dystrophy Type 2. PLoS ONE.

[78] Cardinali B (2009). Microrna-221 and Microrna-222 Modulate Differentiation and Maturation of Skeletal Muscle Cells. PLoS ONE.

[79] Carrer M (2012). Control of mitochondrial metabolism and systemic energy homeostasis by microRNAs 378 and 378*. Proceedings of the National Academy of Sciences.

[80] Rau F (2011). Misregulation of miR-1 processing is associated with heart defects in myotonic dystrophy. Nat Struct Mol Biol.

[81] Koshkin AA (1998). LNA (Locked Nucleic Acids): Synthesis of the adenine, cytosine, guanine, 5-methylcytosine, thymine and uracil bicyclonucleoside monomers, oligomerisation, and unprecedented nucleic acid recognition. Tetrahedron.

[82] Nielsen CB (1999). The Solution Structure of a Locked Nucleic Acid (LNA) Hybridized to DNA. Journal of Biomolecular Structure and Dynamics.

[83] Hutvágner G (2004). Sequence-Specific Inhibition of Small RNA Function. PLoS Biol.

[84] Krutzfeldt J (2005). Silencing of microRNAs in vivo with ‘antagomirs’. Nature.

[85] Ørom UA, Kauppinen S, Lund AH (2006). LNA-modified oligonucleotides mediate specific inhibition of microRNA function. Gene.

[86] Ebert MS, Neilson JR, Sharp PA (2007). MicroRNA sponges: competitive inhibitors of small RNAs in mammalian cells. Nat Meth.

[87] Tay FC (2014). Using artificial microRNA sponges to achieve microRNA loss-of-function in cancer cells. Advanced Drug Delivery Reviews.

[88] Chen L, Zhang K, Shi Z, Zhang A, Jia Z, Wang G, Pu P, Kang C, Han L (2014). A lentivirus-mediated miR-23b sponge diminishes the malignant phenotype of glioma cells in vitro and in vivo. Oncology Reports.

[89] Winbanks CE (2013). miR-206 Represses Hypertrophy of Myogenic Cells but Not Muscle Fibers via Inhibition of HDAC4. PLoS ONE.

[90] Tan SB (2014). Small Molecule Inhibitor of Myogenic microRNAs Leads to a Discovery of miR-221/222-myoD-myomiRs Regulatory Pathway. Chemistry & Biology.

[91] da Costa Martins PA (2010). MicroRNA-199b targets the nuclear kinase Dyrk1a in an auto-amplification loop promoting calcineurin/NFAT signalling. Nat Cell Biol.

[92] Bernardo BC (2014). Therapeutic silencing of miR-652 restores heart function and attenuates adverse remodeling in a setting of established pathological hypertrophy. The FASEB Journal.

[93] Kota J (2009). Therapeutic microRNA Delivery Suppresses Tumorigenesis in a Murine Liver Cancer Model. Cell.

[94] Yang X (2010). Inhibition of hepatitis C virus replication using adeno-associated virus vector delivery of an exogenous anti–hepatitis C virus microrna cluster. Hepatology.

[95] Quattrocelli M (2013). Long-Term miR-669a Therapy Alleviates Chronic Dilated Cardiomyopathy in Dystrophic Mice. Journal of the American Heart Association.

[96] Grimm D (2006). Fatality in mice due to oversaturation of cellular microRNA/short hairpin RNA pathways. Nature.

[97] Odom GL (2008). Microutrophin Delivery Through rAAV6 Increases Lifespan and Improves Muscle Function in Dystrophic Dystrophin/Utrophin-deficient Mice. Mol Ther.

[98] Lai Y (2009). Dystrophins carrying spectrin-like repeats 16 and 17 anchor nNOS to the sarcolemma and enhance exercise performance in a mouse model of muscular dystrophy. The Journal of Clinical Investigation.

[99] Lai Y (2014). Partial restoration of cardiac function with APDZ nNOS in aged mdx model of Duchenne cardiomyopathy. Human Molecular Genetics.

[100] Charan RA (2012). Adeno-associated virus serotype 8 (AAV8) delivery of recombinant A20 to skeletal muscle reduces pathological activation of nuclear factor (NF)-*κ*B in muscle of mdx mice. Molecular Medicine.

[101] Pantazi A, Zovoilis A, Ying SY (2013). Vector-Free Methods for Manipulating miRNA Activity In Vitro and In Vivo. MicroRNA Protocols.

[102] Wallace LM (2012). RNA Interference Inhibits DUX4-induced Muscle Toxicity In Vivo: Implications for a Targeted FSHD Therapy. Mol Ther.

[103] Liu N (2011). Mice lacking microRNA 133a develop dynamin 2-dependent centronuclear myopathy. The Journal of Clinical Investigation.

[104] Clop A (2006). A mutation creating a potential illegitimate microRNA target site in the myostatin gene affects muscularity in sheep. Nat Genet.

